# Horizontal and Vertical Distribution of Heavy Metals in Farm Produce and Livestock around Lead-Contaminated Goldmine in Dareta and Abare, Zamfara State, Northern Nigeria

**DOI:** 10.1155/2017/3506949

**Published:** 2017-05-02

**Authors:** O. E. Orisakwe, O. O. Oladipo, G. C. Ajaezi, N. A. Udowelle

**Affiliations:** ^1^Toxicology Unit, Faculty of Pharmacy, University of Port Harcourt, Rivers State, Nigeria; ^2^National Veterinary Research Institute, Vom, Nigeria; ^3^Department of Medical Laboratory Science, Faculty of Science, Rivers State University of Science and Technology, Port Harcourt, Rivers State, Nigeria

## Abstract

*Background*. Hitherto studies in response to the June 2010 lead poisoning, Zamfara State, Nigeria, have focused on clinical interventions without information on livestock and other metals.* Objective*. This study has investigated the distribution of heavy metals in farm produce and livestock around lead-contaminated goldmine in Dareta and Abare, Zamfara State, Nigeria.* Methods*. Vegetables, soil, water, blood, and different meat samples were harvested from goat, sheep, cattle, and chicken from Dareta, Abare, and Gusau communities. The samples were digested with 10 mL of a mix of nitric and perchloric acids; the mixture was then heated to dryness. Lead, cadmium, zinc, chromium, copper, magnesium, and nickel were analysed using flame Atomic Absorption Spectrophotometer. The daily intake, bioaccumulation factor, and target hazard quotient (THQ) were calculated.* Results*. Chicken bone-muscles from Dareta had the highest concentrations of lead, zinc, and nickel (28.2750, 16.1650, and 4.2700 mg/kg, resp.), while chicken brain had the highest levels of cadmium, magnesium (0.3800 and 67.5400 mg/kg), and chromium (6.1650 mg/kg, kidney tissue inclusive).* Conclusion*. In addition to lead, cadmium may also be of concern in the contaminated mining communities of Zamfara State, Nigeria, given the high levels of cadmium in meat and vegetables samples from these areas.

## 1. Introduction

In early 2010, an epidemic of lead poisoning was discovered during routine meningitis surveillance in Zamfara State, Nigeria, conducted during February–April 2010, Médecins Sans Frontières (MSF) and local public health officials identified more than 200 children (<5 years old) with history of convulsions during the previous 3 months in four villages.

Zamfara State is located in northwestern Nigeria with a population of about four million people (Joint United Nations Environment Programme/Office for the Coordination of Humanitarian Affairs Environment Unit 2010). Although farming is the major livelihood of the local population, Zamfara has been the scene of artisanal gold mining for decades.

Risk assessment, contaminant characterization, and remedial assessment studies done from May 2010 to July 2011 confirmed that the primary exposure route is the incidental ingestion of lead-contaminated soils and household dust [1]. Contamination of food sources during food preparation and processing, inhalation of contaminated dust, and consumption of contaminated water are secondary exposure pathways that magnify lead intake for the exposed populations [1]. Heavy metals precipitate onto topsoil to increase the human health risk, and this risk is then magnified by the accumulation of heavy metals into plants grown on this topsoil. Indeed, vegetables and fruits can contain high levels of heavy metals [2]. In addition to lead, other heavy metals may be present in the ores, including but not limited to cadmium, zinc, chromium, copper, and nickel. Lead is the primary toxicant of concern and remediation removes secondary heavy metals while targeting lead. However, the overall risk to the populations is not limited to lead poisoning. The public health effects of lead exposure to adult and children are documented in literature; however, the combined effects of coexposure to a mixture of heavy metals is largely unknown, especially for intense, chronic exposures.

Lead ingestion or inhalation can cause damage to the brain, kidneys, bone marrow, and other body systems in young children. In infants and children, blood lead levels (BLLs) as low as 5 *μ*g/dL have been associated with developmental problems, including impaired cognitive function, behavioral difficulties, impaired hearing, and reduced stature [3]. BLLs ≥75 *μ*g/dL can cause coma, convulsions, and death. From studies on pre- and postnatal cadmium exposure and IQ deficits, cadmium has been reported to be a potential neurotoxicant. Developmental exposure in laboratory animals indicates that operant performance and conditioned avoidance are negatively impacted as well [4]. Cadmium appears to cross the placental barrier and accumulate in the foetus resulting in neurodevelopmental toxicity [5].

Nickel is an essential trace element in animals, although the functional importance of nickel has not been clearly demonstrated. Although other heavy metals may be involved, nickel seems to be implicated in chronic bronchitis, emphysema, pulmonary fibrosis, and impaired lung function [6]. Copper and chromium are very important essential elements in man but excessive intakes have long been associated with toxicity [7]. Copper is a component of enzymes in iron metabolism and deficiency of this element causes anaemia [7]. Chromium helps to maintain normal blood glucose level and is widely used in diabetes medications [8]. Toxicity of copper and chromium occurs in both acute and chronic forms in cases of excessive intakes [9–11]. Acute copper toxicity signs are nausea, vomiting, jaundice and liver necrosis, damage to the proximal tubules of the kidney, and haemolytic anaemia [9–11]. Wilson's disease in man is a form of chronic copper toxicity with clinical signs including mental alterations, motor abnormalities, dysphagia, ataxia, haemolytic anaemia, renal dysfunction, renal stones, and hepatic failure [9]. Normally chromium toxicity is due to physical contact with contaminated dust or soil resulting in allergic dermatitis characterized by eczema [10].

Whereas most studies in response to the Zamfara lead poisoning have been centered on clinical intervention and reduction of blood lead level (BLL) in children, [12] highlighted the limitations as follows: nonassessment of lead poisoning in livestock in other foods including dairy products. Since many villagers may be exposed through consumption of lead-contaminated foods, [12] recommended the need to characterise the depth and extent of lead distribution in livestock. It is also known that lead does not exist alone in the earth crust and several cases of lead intoxication occur as a cocktail of heavy metal mixture. The present study is aimed at addressing the limitations of [12] study with an investigation into the horizontal and vertical distribution of heavy metals in farm produce and livestock around lead-contaminated goldmine in Dareta and Abare, Zamfara State, Northern Nigeria.

## 2. Materials and Methods

**Figure 1:**
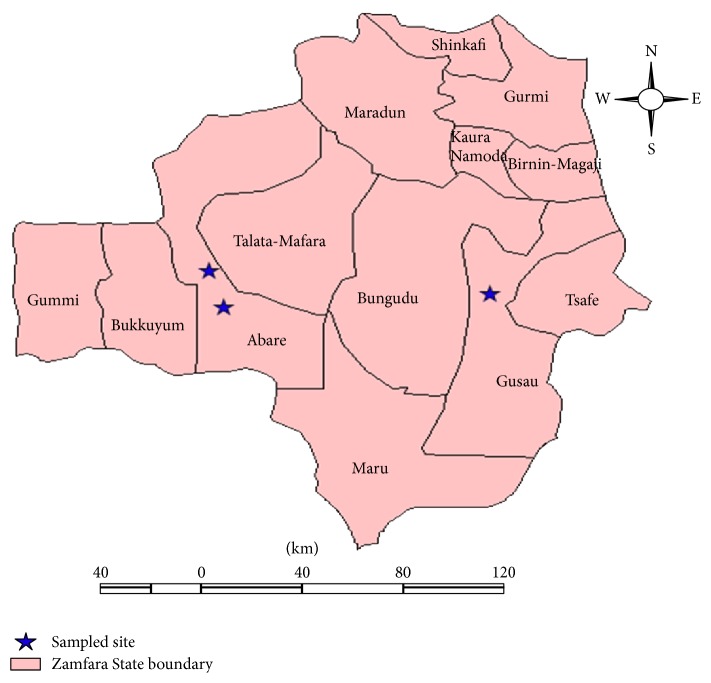
Map of Zamfara State showing local government areas and sampling sites.

A cross-sectional study was carried out in June 2010 in Zamfara State, Nigeria. After focus group discussions with community heads, representatives of the pastoralist, gold miners/processors, the following sampling sites (Dareta, Abare, and Gusau; Figure 1) were chosen. Two hundred milligrams of liver, spleen, kidney, lungs, skeletal muscle, and brain of cattle, sheep, goat, and chicken were harvested. Blood samples (5 mls) of cattle, sheep, goat, and chicken were also taken. Vegetable, pasture soil, and water (waste and drinking) were also collected for lead, cadmium, zinc, chromium, copper, magnesium, and nickel analyses.

### 2.1. Study Area and Sampling

#### 2.1.1. Sample Preparation

Samples were first washed with deionised water. All samples were oven-dried at 70–80°C for 24 hours. Dried samples were ground using a pestle and mortar and sieved through muslin cloth. The protocol was approved by the Research Ethical Committee of the University of Port Harcourt, Rivers State, Nigeria.

#### 2.1.2. Sample Digestion

Five milliliters each of the water samples was acidified to the level of pH 2 with nitric acid.

For each tissue sample or grass, 0.2 g each was weighed and placed in crucibles for ashing in a furnace for 4 h at 550°C. The ash was digested with perchloric acid and nitric acid (1 : 4, v : v) solution. The samples were left to cool and made up to a final volume of 25 ml with deionised water. The hydrolysed samples were well shaken and centrifuged at the rate of 3000*g* to remove solid particles. The resulting homogenized samples were vortex mixed before subsamples were taken for analysis to ensure the homogeneity of the mixture.

The presence of lead, cadmium, zinc, chromium, copper, magnesium, and nickel was analysed using flame Atomic Absorption Spectrophotometer according to the specification of the manufacturer (Shimadzu AAS 6800 B Model). The limits of detection for the toxic metals were all 0.005 ppm with blank values reading as 0.00 ppm for all the metals in deionised water with electrical conductivity value lower than 5 *μ*S cm^−1^.

### 2.2. Quality Control

Appropriate quality procedures and precautions were carried out to assure the reliability of the results. The reagents used to calibrate the instrumentation were of analytical grades. Quality assurance measures were taken to reduce the risk of contamination in order to ensure the reliability of data.

## 3. Data Analyses

### 3.1. Daily Metals Intake Estimate

The daily intake was calculated by the following equation [23]:

(1) $$\begin{array}{c} \text{DMI (mg/day)}=\frac{C_{\text{metal}}\times D_{\text{intake}}}{B_{\text{weight}}}, \end{array}$$

where *C*_metal_ is the metal concentration in sample taken for analysis (in mg/kg). *B*_weight_ is the body weight (6 kg in this study). *D*_intake_ is the daily intake (0.345 kg/person/day) [24, 25].

### 3.2. Target Hazard Quotient (THQ) and Total THQ

Target hazard quotients were developed by the Environmental Protection Agency (EPA) in the US for the estimation of potential health risks associated with long-term exposure to chemical pollutants [26]. This includes not only the intake of metals but also another significant data as exposure frequency and duration, body weight, and the oral reference dose (RfD). The THQ is a ratio between the measured concentration and the oral reference dose (RfD), weighted by the length and frequency of exposure, amount ingested, and body weight. The THQ <1 means the exposed population is assumed to be safe and 1 < THQ < 5 means that the exposed population is in a level of concern interval. THQ parameter is a dimensionless index and THQ values are additive, but not multiplicative. It must be noted that THQ is not a measure of risk but indicates a level of concern. THQ was determined based on the formula (modified) given by [27]:

(2) $$\begin{array}{c} \text{THQ}=\left(\;\frac{\text{EFr}\times {\text{ED}}_{\text{tot}}\times \text{FIR}\times \text{C}}{{\text{RfD}}_{\text{o}}\times {\text{BW}}_{\text{a}}\times {\text{AT}}_{\text{n}}}\;\right)\times 10^{-3}, \end{array}$$

where the parameter values are the following: EFr, exposure frequency = 365 days/year; ED_tot_, exposure duration = 70 years, equivalent to average lifetime [28]; FIR, food ingestion rate = 0.345 kg/person/day [24, 25]; *C*, concentration of metal in animal tissue in mg/kg; RfD_o_, oral reference dose in mg/kg/day; BW_a_, average body weight, adult = 60 kg; AT_n_, average exposure time for noncarcinogens in days (EFr (365 days/year) ×  ED_tot_ (number of exposure years, assuming 70 years in this study); 10^−3^, the unit of conversion).

The total THQ (TTHQ) of heavy metals for the individual parameter assayed was calculated according to [27] as the sum of the individual THQ of the heavy metals.

The total THQ is the sum of the following compositions:

(3) Total  THQ  TTHQ=THQ  toxicant  1+THQ  toxicant  2+⋯+THQ  toxicant  n.

The data were also analysed using GraphPad InStat version 6.03 statistical package to obtain the mean, standard deviation, standard error of the mean, range, and the linear correlation between the measured parameters.

### 3.3. Bioaccumulation Factor (BAF)

The bioaccumulation factor (BAF), an index of the ability of the vegetables to accumulate a particular metal with respect to its concentration in the soil substrate, was calculated as follows:

(4) $$\begin{array}{c} \text{BAF}=\frac{C_{\text{grass}}}{C_{\text{soil}}}, \end{array}$$

where *C*_grass_ and *C*_soil_ represent the heavy metal concentration in grass and soil, respectively.

## 4. Results

Table 1 gives the mean blood levels of the heavy metals, namely, lead, cadmium, zinc, chromium, copper, magnesium, and nickel in the sampled goat, sheep, cattle, and chicken, and the levels of individual sample variability from one another as well as dispersion from sampling size. Goat and chicken (blood) from Dareta had the highest mean concentrations of lead and cadmium (7.7554 and 0.3258 mg/kg, resp.), while cattle from Abattoir had the highest mean blood levels for zinc (2.0400 mg/kg), chromium (3.2375 mg/kg), and copper (1.4788 mg/kg). Goat from Abare had the mean maximum values for magnesium and nickel (19.6133 and 3.9583 mg/kg, resp.). Goats from Dareta had the highest amounts of blood lead and copper (30.2750 and 3.1650 mg/kg), and chicken from Dareta had blood cadmium (0.7200 mg/kg), while cattle from Abattoir had the highest blood levels of both zinc and chromium (5.5980 and 7.6700 mg/kg, resp.) (table not shown). Goats from Abare had the highest blood levels of magnesium and nickel (20.4950 and 6.7200 mg/kg, resp.) (table not shown). The blood levels of lead in goats from Dareta and Abare are 7.7554 and 5.0867 mg/kg, respectively. Sheep and cattle from Dareta had blood lead levels of 6.2550 and 5.755 mg/kg, respectively (table not shown).

Table 2 presents the mean concentrations of the heavy metals lead, cadmium, zinc, chromium, copper, magnesium, and nickel in some of the environmental matrices (vegetable, plant, soil, and water) and the levels of individual sample variability from one another as well as dispersion from sampling size. The minimum and the maximum values are also indicated in form of ranges. The mean concentrations of magnesium in both the vegetable and water samples were the most elevated (68.463 and 17.836 mg/kg, resp.). Soil sample had the highest mean concentration of lead (46.039 mg/kg). Vegetable from Dareta had the most elevated levels of lead, copper, and magnesium (102.8350, 23.8150, and 72.0950 mg/kg, resp.), while soil samples from Dareta (cadmium and chromium, 0.5000 and 40.2500 mg/kg) and Abare (zinc and nickel, 8.6400 and 4.3750 mg/kg) had the highest concentrations, respectively (not shown in the table).

The oral reference doses (RfD), the highest estimated daily intake, and upper tolerable daily intakes for metals (UL) are presented in Table 3. No RfD has been set by USEPA for magnesium.

Table 4 shows the levels of the heavy metal intake through the daily consumption of the various animal compartments. The mean daily intakes for lead, cadmium, zinc, chromium, copper, magnesium, and nickel for chicken liver tissues from Dareta were 0.0214, 0.0013, 0.0283, 0.0147, 0.0058, 0.1878, and 0.0058 mg/day, respectively, with LChD1 having the highest daily intake for Pb (0.0460 mg/day). LChD2 had the highest intake for Cd at 0.0020 mg/day. LChD3 has the maximum intakes for chromium (0.0268 mg/day) and nickel (0.0132 mg/day), while the highest intakes for Zn and Mg were seen in LChD6. LChD5 had the highest daily intake for Cu at 0.0082 mg/day. For cattle liver tissues from Abattoir, LCAb9 had the maximum daily intake for lead (0.0317 mg/day), cadmium (0.0031 mg/day), and nickel (0.0315 mg/day). LCAb13 the highest intake for zinc (0.0490 mg/day), LCAb12 had the maximum intake for chromium (0.0231 mg/day) and copper (0.0412 mg/day), and LCAb11 had the highest intake for magnesium (0.1730 mg/day). The mean daily intakes for Pb, Cd, Zn, Cr, Cu, Mg, and Ni if cattle liver tissues from Abattoir are consumed were 0.0163, 0.0014, 0.0240, 0.0181, 0.0199, 0.1519, and 0.0177 mg/day, respectively. The daily intake rate for chicken kidney from Dareta was Pb (0.0259 mg/day), Cd (0.0017 mg/day), Zn (0.0175 mg/day), Cr (0.0354 mg/day), Cu (0.0063 mg/day), Mg (0.1505 mg/day), and Ni (0.0123 mg/day), while that for chicken muscle was Pb (0.0417 mg/day), Cd (0.002 mg/day), Zn (0.0118 mg/day), Cr (0.0271 mg/day), Cu (0.0039 mg/day), Mg (0.1433 mg/day), and Ni (0.0063 mg/day). The mean Pb daily intakes for chicken bone-muscle, lungs, intestine, and brain from Dareta were 0.1144, 0.0245, 0.0123, and 0.0504 mg/day, respectively. For Cd, the mean daily intakes were 0.0014, 0.0013, 0.0005, and 0.0022 mg/day, respectively, while for Zn, they were 0.0723, 0.0245, 0.0161, and 0.0207 mg/day, respectively. Cu had mean daily intakes of 0.0060, 0.0048, 0.0216, and 0.0271 mg/day, respectively, and Mg 0.3453, 0.1759, 0.1635, and 0.2530 mg/day, respectively. The average daily intakes for Ni were 0.0144, 0.0129, 0.0096, and 0.0158 mg/day, respectively. Since the concentrations of Cr in both chicken bone-muscle samples from Dareta were 0.001 mg/kg, the daily intake is negligible. For chicken lungs, intestine, and brain from Dareta, the average daily intake for Cr were 0.0196, 0.0260, and 0.0278, respectively.

The target hazard quotient (THQ) and total target hazard quotient (TTHQ) calculations were used to assess the potential health risk in the consumption of the different animal tissues. These THQs were calculated using the oral reference doses (mg/kg/day) (Cr—1.5; Ni—2.0 × 10^−2^; Pb—4.0 × 10^−3^; Cd—1.0 × 10^−3^; Cu—4.0 × 10^−2^; Zn—3.0 × 10^−1^) of the individual metal as stipulated by USEPA (USEPA, IRIS, 1997, 2007). The THQ and TTHQ for all the metals were all below 1.0 (table not shown).

The metal levels in blood and liver showed significant correlation, while Cd and Cu showed a negative correlation. There were significant correlations in Mg and Ni (*r* = 0.7983 and 0.6143, resp.) in blood and kidney, bone-muscle, muscle, lung, intestine, and brain. Lead, chromium, and copper were found to be negatively correlated. Chromium, cadmium, and copper were strongly correlated (*r* = 0.7845, 0.6879, and 0.9058, resp.). Only chromium and zinc showed weak correlation in water and grass samples.

**Figure 2:**
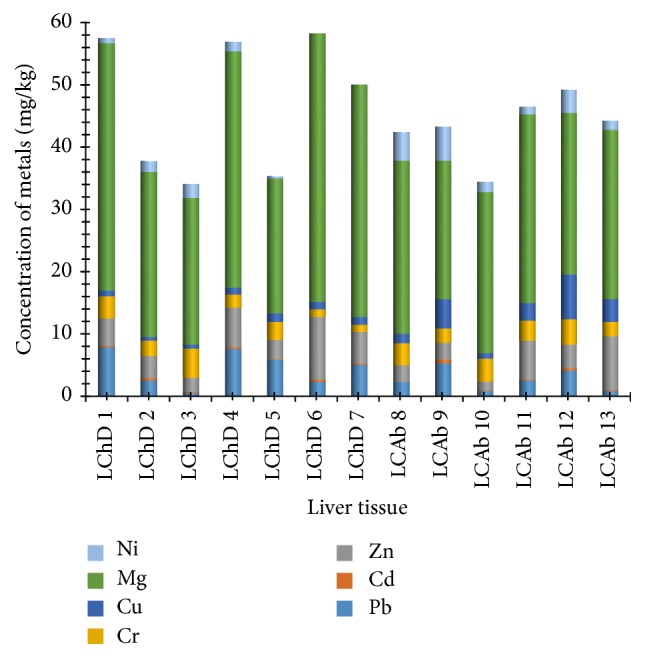
The levels (mg/kg) of heavy metals in liver tissues (chicken and cattle) from Dareta and Abattoir. L: liver tissue; C: cattle; Ch: chicken; D: Dareta; Ab: Abattoir.

Figure 2 shows the different concentrations of the heavy metals lead, cadmium, zinc, chromium, copper, cobalt, magnesium, and nickel in the liver tissues of chickens and cattle. Liver tissues of chickens from Dareta had the highest amounts of lead, zinc, chromium, and magnesium (8.0050, 9.9700, 4.6550, and 42.9350 mg/kg, resp.), while cattle from Abattoir had the highest levels of both copper and nickel (7.1600 and 5.4700 mg/kg, resp.). The average levels of lead, cadmium, zinc, chromium, copper, magnesium, and nickel in chicken liver tissues from Dareta are 4.7171, 0.2336, 4.9229, 2.5507, 1.0121, 32.6586, and 1.0121 mg/kg, respectively, while the mean concentrations of the respective metals in cattle liver tissues from Abattoir are 2.8350, 0.2500, 4.1792, 3.1450, 3.4533, 26.4067, and 3.0642 mg/kg. Magnesium was the highest in all liver samples. Other metals had varying concentrations in the samples, with sample LCAb8 showing a negligible level of cadmium. The levels of nickel in samples LChD6 and LCHD7 were also negligible.

**Figure 3:**
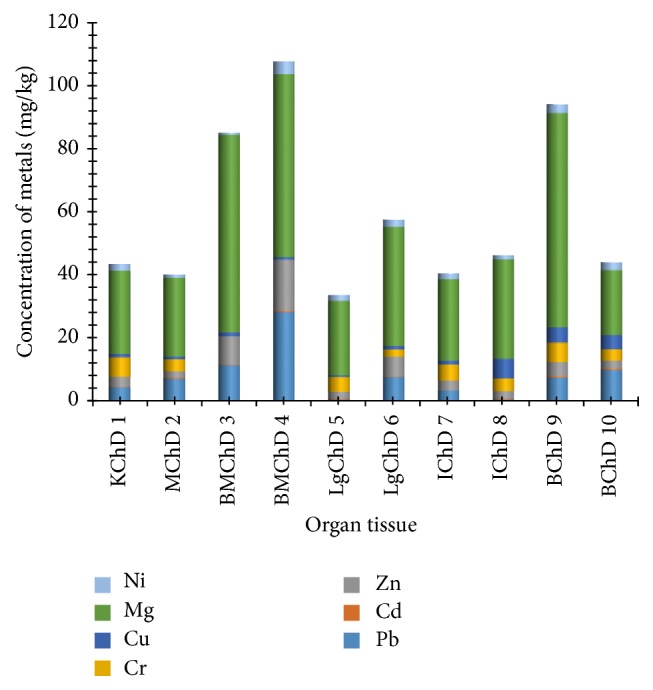
The levels (mg/kg) of heavy metals in kidney, muscle, bone-muscle, lung, intestine, and brain tissues in chicken from Dareta. K: kidney; M: muscle; BM: bone-muscle; Lg: lung; I: intestine; B: brain; Ch: chicken; D: Dareta.

Figure 3 shows the different concentrations of the heavy metals lead, cadmium, zinc, chromium, copper, magnesium, and nickel in the kidney, muscle, bone-muscle, lung, intestine, and brain tissues. Chicken bone-muscles from Dareta had the highest concentrations of lead, zinc, and nickel (28.2750, 16.1650, and 4.2700 mg/kg, resp.), while chicken brain tissues had the highest levels of cadmium, magnesium (0.3800 and 67.5400 mg/kg), and chromium (6.1650 mg/kg, kidney tissue inclusive). The intestine had the most elevated copper level (6.2250 mg/kg). BMChD4 had the highest cumulative concentration of the heavy metals, followed by samples BChD9 and BMChD3, respectively. Sample LgChD5 had the lowest cumulative level of the heavy metals. The levels of magnesium were seen to be high in all organ-tissue samples.

**Figure 4:**
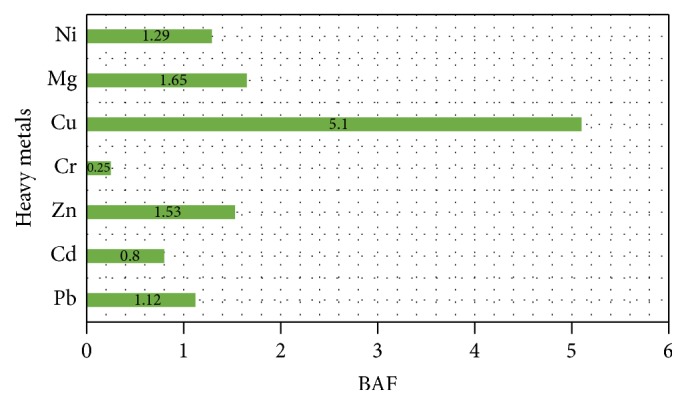
Bioaccumulation factor in vegetables.

The metal pollution index in vegetables is shown in Figure 4. Lead and nickel exceeded the metal pollution of one while the MPI of cadmium was also high.

## 5. Discussion

Artisanal mining is an essential activity in many developing countries as it provides an important source of livelihood, particularly in regions where economic alternatives are limited. Unfortunately, these activities are frequently accompanied by extensive environmental degradation and deplorable social conditions, both during operations and well after mining activities have ceased. Potential toxic metals are natural components of the environment, but human activities, notably industrial mining processes, have been responsible for the wider distribution of these elements. Mining and processing metal ore can be a significant source of heavy metal contamination of the environment [29]. The environmental concern in mining areas is primarily related to physical disturbance of the surrounding landscape, spilled mine tailings, emitted dust, and acid mine drainage transported into rivers. Excessive accumulation of heavy metals in agricultural soils around mining areas, resulting in elevated heavy metal uptake by food crops, is of great concern because of potential health risk to the local inhabitants [30]. The consumption of foods produced in contaminated areas and the ingestion or inhalation of contaminated particles are two principal exposure pathways of these potential toxic metals in man. Potential health risks to humans and animals from consumption of crops can be due to heavy metal uptake from contaminated soils via plant roots as well as direct deposition of contaminants from the atmosphere onto plant surfaces [31]. Food contamination by heavy metals depends on both their mobility in the soil and their bioavailability. Though some of the mobility and bioavailability factors are easy to measure, determination of the food risk contamination is tricky. The lead and cadmium levels in soil and vegetable from Dareta are higher than the values reported by [32]. Lead and cadmium levels in vegetables grown in irrigated farmland of Kaduna metropolis have been reported to be 0.92–4.67 *μ*g Pb/g and 0.1–1.3 *μ*g Cd/g [32]. Consumption of the vegetables from this region may constitute health hazard to humans since these metals' levels were above FAO/WHO alimentarium standard. Metal pollution index (MPI) was calculated in this study to determine the metal pollution monitoring in soil and vegetable. The impact of heavy metal pollution on different matrices depends on the bioaccumulation of the individual metals which are toxic at high concentrations. Pollution index calculates the relative contamination of different metals separately and manifests the sum of generated components as a representative [33]. A heavy metal pollution index value of >1 indicates pollution, whereas MPI value of <1 indicates no pollution [34]. The MPI values of lead, nickel, and cadmium, which were all <1 in the vegetable, are an evidence of significant contamination of both human and veterinary public health importance.

Although the THQ and TTHQ for all the metals were all below 1.0, suggesting no metallotoxicity from the ingestion of these meat, vegetables, and drinking water from Zamfara State artisanal mining communities, potential chronic toxicity is of concern given the fact that some of the metals exceeded the maximum allowable limits in foods. The accumulation of cadmium, copper, magnesium, and nickel in the liver of goat, sheep, cattle, and chicken may be independent of their blood levels as suggested by their poor** c**orrelation. The significant correlation in Mg and Ni (*r* = 0.7983 and 0.6143, resp.) in blood and kidney, bone-muscle, muscle, lung, intestine, and brain suggests that increases in the blood levels of Mg and Ni may have contributed to their accumulation in the kidney, bone-muscle, muscle, lungs, intestine, and brain of the animals. Similarly, the soil sample concentrations of cadmium, chromium, and copper may have contributed to the accumulation of these metals in the vegetable samples as suggested by their strong correlation. The contamination of soils with toxic environmental contaminants is a pervasive problem of potential human health concern to those individuals working and residing near hazardous waste sites. Soil contamination may directly impact human health through the following exposure pathways: incidental ingestion of surface soil, particularly by children who may gain access to the site or by on-site workers exhibiting frequent hand-to-mouth activity (e.g., smokers); and direct dermal contact and inhalation of contaminated fugitive dust by workers and nearby residents during remedial activities or inclement weather conditions. In addition, soil contamination may adversely impact human health by contributing to groundwater and surface water contamination by means of infiltration and surface runoff, respectively. Artisanal mining activity from the present study tended to have negligible impact of the levels of lead, cadmium, zinc, chromium, and copper in the drinking water. Drinking water might not be a major source of metal pollution in Zamfara.

The potential toxic metals, lead, cadmium, nickel, zinc, chromium, and copper, are, respectively, classified as numbers 2, 7, 57, 75, 78, and 118 on the priority list of the most hazardous substances in the environment by the Agency for Toxic Substances and Diseases Registry [35]. Some metals such as cadmium are mobile in plants and may become concentrated in leaves. Grazing animals may potentially be highly exposed to these elements via ingestion of polluted soil, vegetation, and drinking water and possibly also via inhalation [36, 37]. Knowledge of metal concentrations in livestock is therefore important for assessing the effects of these pollutants on domestic animals, as well as the potential risk from human consumption.

Lead poisoning is more common in farm ruminants [38]. In ruminants blood lead level up to 0.25 *μ*g/mL is considered safe; however, BLL above 0.35 *μ*g/mL is toxic [39]. The higher BLLs seen in goats, sheep, and cattle reared around these mining sites may be mainly due to ingestion of pasture contaminated with lead as well as inhalation of lead particles [40]. Lead is considered as the most common form of poisoning in farm animals because of natural curiosity, licking habits, and lack of oral discrimination [41]. In this study, BLLs of goat (2.0000–30.2750 mg/kg) (1.7500–7.2550) in Dareta and Abare, respectively, sheep (3.0000–9.5100 mg/kg), cattle (0.7500–10.7600 mg/kg), and chicken (0.7500–8.2550 mg/kg) in Dareta, reared in mining sites, were higher than 0.35 *μ*g/mL, toxic level reported for ruminants [39]. Chicken meat from Dareta had lead levels ranging from 0.7500 to 28.2750 mg/kg, chromium levels of up to 6.1650 mg/kg, and nickel levels from 0.7300 to 4.2700 mg/kg. Lead levels exceeded the Codex Alimentarius guideline for Pb in muscles (0.1 mg/kg) and edible offal (0.5 mg/kg) of cattle.

In a study of cadmium and lead levels in the muscle and edible offals of cow reared in Nigeria, [42] reported cadmium (mg/kg) which ranged from 0.01–0.80 in muscle, <0.004–0.90 in liver, 0.10–1.12 in kidney, 0.01–0.90 in intestine, and 0.01–1.10 in tripe while the lead was <0.005–0.72 in muscle, 0.08–501.79 in liver, 0.04–44.89 in kidney, 0.01–108.02 in intestine, and 0.01–127.90 in tripe. Similarly high cadmium levels in kidney (0.07–3.08 *μ*g/g) and in muscle (0.09–1.26 *μ*g/g) have been reported by [43] in goat meat in Nigeria. Paper [44] reported high lead level (59.03–88.88 *μ*g/g in liver; muscle (47.36–147.07 *μ*g/g)) and high cadmium level (liver (1.78–8.80), muscle (6.53–15.32 *μ*g/g), and gizzard (3.78–7.10 *μ*g/g)) in local and exotic poultry meat. Paper [45] reported mean lead levels (*μ*g/g): 66.70 ± 0.70 in lungs; 85.20 ± 0.00 in liver, and 38.70 ± 0.20 in kidney of cow in Kwara State, Nigeria. Paper [46] reported Pb (0.01–4.60 mg/kg) and Cd (0.01–5.68 mg/kg) in chicken meat from southern Nigeria. Paper [47] reported high levels of Pb (0.80–1.42 mg/kg), Cd (0.28–1.50 mg/kg), Pb (0.02–1.36 mg/kg), and Cd (0.04–0.93 mg/kg) in cow and goat meat parts, respectively. In all, different anthropogenic activities ranging from burning of fossil fuel, vehicular traffic, and so forth were attributed as likely sources of these metals.

The high potential toxic metal levels in these meat samples must have been due to the artisanal mining activities. The presence of high levels of potential toxic metals in all the meat samples in this study is of grave public health importance.

Blood cadmium level in chicken from Dareta was the highest. Cadmium levels in some of the meat samples exceeded the EU set limit of 0.05 mg/kg for Cd in meat.

The mean cadmium levels in the goat, sheep, cattle, and chicken meat parts from the contaminated areas of Zamfara State are higher than 0.161 mg/kg reported by Miranda and coworkers in 2005 in cattle from polluted industrialised communities of Spain. From many polluted areas around Europe, different meat cadmium levels have been documented, namely, 0.038 mg/kg [48] of meat from polluted area in Poland; 0.021 mg/kg [49] in Finland; 0.053 mg/kg [50] in Hungary; and 0.0985 mg/kg [51] in Eastern Slovakia. Taken together, the mean meat cadmium levels from Zamfara in this study were higher than the European values but were however lower than 1.6 mg/kg (fresh weight) that Garco-Rico and coworkers [52] reported in swine kidney from Sonora. Cadmium has a long biological half-life of about 30 years in humans [53]. It damages the proximal tubules of nephron, leading first to proteinuria and essential ions like calcium into the urine which may progress to renal failure [54]. Cadmium has been shown to be toxic to human populations from occupational inhalation exposure and accidental ingestion of cadmium-contaminated food. Inhalation of cadmium dust in certain occupational settings may be associated with an increased incidence of lung cancer. Other symptoms include irritation of upper respiratory tract, metallic taste in the mouth, cough, and chest pains [55, 56]. Cadmium is poorly absorbed from the lung, gastrointestinal tract, and skin. Individuals with dietary deficiencies of iron, calcium, or protein exhibit higher absorption of ingested cadmium. Cadmium in the body binds readily to certain sulphur-containing proteins, such as metallothionein. Binding to metallothionein is thought to reduce the toxicity of cadmium. Following ingestion, faecal excretion is high due to poor gastrointestinal absorption. Most cadmium that has been absorbed, however, is excreted very slowly, with faecal and urinary excretion being about equal. Urinary cadmium levels are an indicator of body burden. Experimental studies and epidemiological surveys have linked cadmium exposure with many types of cancer such as lung, prostate, renal, liver, hematopoietic system, urinary bladder, pancreatic, testis, and stomach cancers [57–59]. Exposure to cadmium can also affect the function of the nervous system [60, 61], with symptoms including headache and vertigo, olfactory dysfunction, Parkinsonian-like symptoms, slowing of vasomotor functioning, peripheral neuropathy, decreased equilibrium, decreased ability to concentrate, and learning disabilities [62, 63].

Copper is a trace element needed in the body for various biochemical processes with permissible limits of 20 mg/day and a lethal dose limit of 100 mg/day. The amount of copper in most food is usually about 5 to 7 mg/kg; therefore, cases of copper poisoning are rarely reported [64]. The maximum levels of the estimated daily intake of all the metals in the analysed meat samples were all below their respective permissible limits or upper tolerable limits as stipulated by the various authorities. Copper and zinc levels were low in all the goat, sheep, cattle, and chicken parts including muscles in this study. Muscle is one of the important tissues for zinc accumulation and possesses zinc concentrations that were similar to those in the liver [65]. The Cd/Zn ratios in goat, sheep, and chicken meat from Dareta, the most polluted community, were 0.16, 0.15, 0.43, and 0.46, respectively. Thus, the low zinc concentration in the different meat samples may be attributed to higher cadmium accumulation in these meat samples. The high levels of the potential toxic metals including cadmium in the goat, sheep, cattle, and chicken parts from the artisanal mining areas of Zamfara State, Nigeria, tended to impair the meat quality. According to [66, 67], cadmium causes reductions in both intestinal zinc absorption and hepatic zinc reserves in cattle, respectively, as a result of competition for the cation-binding sites of metallothionein (MT).

The highest estimated daily intake of nickel in this study was 0.0315 (mg/kg bw day). The World Health Organization (WHO) tolerable daily intake (TDI) of nickel is 5 *μ*g/kg bw day. Higher dietary nickel contributions, ranging from 200 to 900 *μ*g day-1, have been reported [68, 69]. The dietary intake of nickel does not lead to any health risk in the general population but could be of public health concern in some sensitized individuals [70]. The lung has been identified as the critical target of nickel toxicity. Nickel substitution for other essential elements may contribute to the adverse health effects of nickel. Nickel can replace magnesium in certain steps in the activation of complement [71]. The replacement of nickel for magnesium leads to a 40-fold increase in the formation of C3b, Bb enzyme, which amplifies activation of the complement pathway.

Concurrent exposure to heavy metal mixtures near mining sites is well known and environmental health issues in developing nations are more often exacerbated by socioeconomic and political instabilities. The scenario of increasing chemical disease combined with morbidity due to communicable disease exacerbates the already beleaguered public health problems of developing nations [72]. As the incidence of hazardous waste sites, pesticide and pharmaceutical production, manufacturing enterprises, and mining become more prevalent in developing countries, environmental health crises will increasingly contribute to environmental and public health burden. Small-scale mining grew by 20% in a five-year period in 35 countries across Africa, Asia, and Latin America [73]. Studies of toxicological interactions of chemical mixtures predominantly focus on pairs of xenobiotics [74]. Yet the relevance of multiple toxicant exposures is critical in global public health. The present study has therefore shown that lead may not be the only toxic metal of public health concern in the contaminated mining communities of Dareta, Abare, and Gusau of Zamfara State given the high levels of cadmium in meat and vegetables samples from these areas. In view of the nonbiodegradable and persistent nature of heavy metals and the long-term low dose exposure of the population to these heavy metals, the associated health hazards should be investigated to understand the size of the problem. To prevent health risks, government and relevant regulatory agencies should adopt more stringent measures to reduce and monitor heavy metal contamination of soil and agricultural produce and livestock [75]. Mining activities which ceased decades or even centuries ago but worked under the absence of environmental health regulations constitute significant source of contamination and environmental risk [75]. Since there is a marked risk of lead poisoning in younger animals and in cattle herds [76], further studies are imperative to understand the combined impact of these metals especially lead and cadmium in both livestock and man. As a consequence, land remediation and/or alternative management practices are required in these mining communities to reduce the impact of artisanal gold mining activities.

## Figures and Tables

**Table 1:** Blood levels (mg/kg) of heavy metals in goat, sheep, cattle, and chicken from Dareta, Abare, and Abattoir.

Element	Animal	Location	*N*	Mean ± SD	Range (Min–Max)
Pb	Goat	Dareta	13	7.7554 ± 7.4943	2.0000–30.2750
		Abare	3	5.0867 ± 2.9326	1.7500–7.2550
	Sheep	Dareta	2	6.2550 ± 4.6033	3.0000–9.5100
	Cattle	Dareta	2	5.7550 ± 7.0781	0.7500–10.7600
		Abattoir	4	4.7538 ± 5.4933	0.5000–12.5100
	Chicken	Dareta	6	4.4608 ± 3.1066	0.7500–8.2550

Cd	Goat	Dareta	13	0.2835 ± 0.1446	0.0900–0.6150
		Abare	3	0.2433 ± 0.1589	0.1300–0.4250
	Sheep	Dareta	2	0.2150 ± 0.1344	0.1200–0.3100
	Cattle	Dareta	2	0.2950 ± 0.3323	0.0600–0.5300
		Abattoir	4	0.2363 ± 0.2299	0.0000–0.5150
	Chicken	Dareta	6	0.3258 ± 0.2001	0.1750–0.7200

Zn	Goat	Dareta	13	1.7908 ± 1.5463	0.0000–5.3600
		Abare	3	1.8233 ± 0.8498	0.8650–2.4850
	Sheep	Dareta	2	1.4125 ± 0.2793	1.2150–1.6100
	Cattle	Dareta	2	0.7050 ± 0.9334	0.0450–1.3650
		Abattoir	4	2.0400 ± 2.4436	0.0000–5.5900
	Chicken	Dareta	6	0.7217 ± 0.2481	0.4450–1.0450

Cr	Goat	Dareta	13	2.9219 ± 1.1640	0.8800–5.2850
		Abare	3	2.7683 ± 0.5477	2.1400–3.1450
	Sheep	Dareta	2	2.8925 ± 0.1803	2.7650–3.0200
	Cattle	Dareta	2	2.3275 ± 0.9793	1.6350–3.0200
		Abattoir	4	3.2375 ± 3.4230	0.1250–4.1500
	Chicken	Dareta	6	2.4317 ± 1.5375	0.6300–3.6500

Cu	Goat	Dareta	13	1.1123 ± 0.6844	0.4900–3.1650
		Abare	3	0.9300 ± 0.1440	0.8300–1.0950
	Sheep	Dareta	2	1.1300 ± 0.5869	0.7150–1.5450
	Cattle	Dareta	2	1.0150 ± 0.0000	—
		Abattoir	4	1.4788 ± 0.3307	1.2450–1.9800
	Chicken	Dareta	6	0.7733 ± 0.2059	0.5300–1.0950

Mg	Goat	Dareta	13	2.9219 ± 1.1640	0.8800–5.2850
		Abare	3	19.6133 ± 1.3247	18.0900–20.4950
	Sheep	Dareta	2	9.6100 ± 13.5906	0.0000–19.2200
	Cattle	Dareta	2	13.8125 ± 8.3615	7.9000–19.7250
		Abattoir	4	19.2600 ± 1.0357	17.7900–20.1300
	Chicken	Dareta	6	2.6058 ± 0.7654	1.5700–3.1250

Ni	Goat	Dareta	13	1.7869 ± 1.6479	0.0500–5.7800
		Abare	3	3.9583 ± 3.0875	0.6250–6.7200
	Sheep	Dareta	2	2.0050 ± 1.5839	0.8850–3.1250
	Cattle	Dareta	2	1.4325 ± 0.4773	1.0950–1.7700
		Abattoir	4	1.1725 ± 1.0281	0.0000–2.5000
	Chicken	Dareta	6	1.9267 ± 0.9005	1.3000–3.7000

**Table 2:** Mean concentrations (mg/kg) of heavy metals in some physical samples.

Element	Environmental matrix	*N*	Mean ± SD	Range (Min–Max)
Pb	Vegetable	5	51.343 ± 42.634	11.010–102.840
	Soil	4	46.039 ± 29.420	5.755–76.065
	Water	6	1.301 ± 2.159	0.040–5.505

Cd	Vegetable	5	0.288 ± 0.069	0.220–0.380
	Soil	4	0.360 ± 0.122	0.250–0.500
	Water	6	0.058 ± 0.102	0.008–0.265

Zn	Vegetable	5	4.593 ± 1.600	3.205–6.910
	Soil	4	3.000 ± 3.802	0.405–8.640
	Water	6	0.228 ± 0.256	0.041–0.575

Cr	Vegetable	5	3.597 ± 1.466	2.010–5.535
	Soil	4	14.655 ± 17.211	4.530–40.250
	Water	6	1.324 ± 2.077	0.141–5.285

Cu	Vegetable	5	10.069 ± 10.450	1.695–23.815
	Soil	4	1.970 ± 1.539	0.530–4.145
	Water	6	0.354 ± 0.483	0.030–1.015

Mg	Vegetable	5	68.463 ± 7.074	55.840–72.095
	Soil	4	41.508 ± 29.73	2.659–70.845
	Water	6	17.836 ± 14.645	0.861–34.920

Ni	Vegetable	5	2.814 ± 0.768	1.720–3.805
	Soil	4	2.174 ± 1.601	0.675–4.375
	Water	6	1.155 ± 1.395	0.105–3.540

**Table 3:** Oral reference doses (RfD), highest estimated daily intake, and upper tolerable daily intakes (UL) for investigated metals.

S/N	Element	RfD (mg/kg/day) [13]	Highest estimated daily intake (mg/day)	UL (mg/day) [references]
1	Pb	0.004	0.1626	0.4 [14] 0.5 [15, 16]
2	Cd	0.001	0.0031	1.0 [14]
3	Zn	0.300	0.0929	40 [17, 18]
4	Cr	1.5	0.0354	0.5 [19]
5	Cu	0.040	0.0412	10 [17, 18]
6	Mg	—	0.3884	350 [20, 21]
7	Ni	0.020	0.0315	7 (children 1–3 years) 40 (adults 19–70 years) [22]

**Table 4:** Daily intake (mg/day) of heavy metals through consumption of the different animal compartments (tissues).

Animal tissue	Pb	Cd	Zn	Cr	Cu	Mg	Ni
LChD1	0.0460	0.0012	0.0252	0.0202	0.0054	0.2272	0.0051
LChD2	0.0158	0.0020	0.0201	0.0145	0.0030	0.1514	0.0105
LChD3	0.0043	0.0010	0.0128	0.0268	0.0035	0.1347	0.0132
LChD4	0.0043	0.0016	0.0361	0.0123	0.0061	0.2170	0.0093
LChD5	0.0345	0.0004	0.0181	0.0159	0.0082	0.1236	0.0027
LChD6	0.0144	0.0020	0.0573	0.0065	0.0074	0.2469	0.0000
LChD7	0.0302	0.0012	0.0286	0.0065	0.0072	0.2137	0.0000
LCAb8	0.0144	0.0000	0.0151	0.0202	0.0087	0.1588	0.0267
LCAb9	0.0317	0.0031	0.0155	0.0130	0.0264	0.1279	0.0315
LCAb10	0.0058	0.0010	0.0079	0.0210	0.0050	0.1476	0.0102
LCAb11	0.0158	0.0007	0.0354	0.0181	0.0167	0.1730	0.0072
LCAb12	0.0245	0.0024	0.0213	0.0231	0.0412	0.1485	0.0216
LCAb13	0.0058	0.0014	0.0490	0.0130	0.0212	0.1553	0.0087
KChD14	0.0259	0.0017	0.0175	0.0354	0.0063	0.1505	0.0123
MChD 15	0.0417	0.0020	0.0118	0.0217	0.0039	0.1433	0.0063
BMChD 16	0.0662	0.0007	0.0516	0.0000	0.0067	0.3591	0.0042
BMChD 17	0.1626	0.0020	0.0929	0.0000	0.0052	0.3315	0.0246
LgChD 18	0.0043	0.0010	0.0128	0.0268	0.0035	0.1347	0.0105
LgChD 19	0.0446	0.0016	0.0361	0.0123	0.0061	0.2170	0.0132
IChD 20	0.0202	0.0002	0.0182	0.0289	0.0074	0.1473	0.0105
IChD 21	0.0043	0.0008	0.0139	0.0231	0.0358	0.1797	0.0087
BChD 22	0.0432	0.0021	0.0264	0.0354	0.0282	0.3884	0.0168
BChD 23	0.0576	0.0022	0.0149	0.0202	0.0260	0.1176	0.0147

L: liver tissue; C: cattle; Ch: chicken; D: Dareta; Ab: Abattoir; K: kidney; M: muscle; BM: bone-muscle; Lg: lung; I: intestine; B: brain.
